# Molecular Profiling of Acute and Chronic Rejections of Renal Allografts

**DOI:** 10.1155/2013/509259

**Published:** 2013-11-04

**Authors:** Hřibová Petra, Honsová Eva, Brabcová Irena, Hrubá Petra, Viklický Ondřej

**Affiliations:** ^1^Transplant Laboratory, Institute for Clinical and Experimental Medicine, Vídeňská 1958/9, 14021 Prague, Czech Republic; ^2^Department of Transplant Pathology, Institute for Clinical and Experimental Medicine, Vídeňská 1958/9, 14021 Prague, Czech Republic; ^3^Department of Nephrology, Institute for Clinical and Experimental Medicine, Vídeňská 1958/9, 14021 Prague, Czech Republic

## Abstract

Both antibody mediated (AMR) and T-cell mediated (TCMR) rejections either acute or chronic represent the main reason for late graft dysfunction. In this study we aimed to evaluate differences in the intrarenal expression patterns of immune system related genes in acute and chronic rejections. Graft biopsies were performed and evaluated according to Banff classification. Using the TaqMan Low Density Array, the intrarenal expressions of 376 genes relating to immune response (B-cell activation, T-cell activation, chemokines, growth factors, immune regulators, and apoptosis) were analyzed in the four rejection categories: chronic AMR, chronic TCMR, acute AMR, and acute TCMR. The set of genes significantly upregulated in acute TCMR as compared to acute AMR was identified, while no difference in gene expressions between chronic rejections groups was found. In comparison with functioning grafts, grafts that failed within the next 24 months after the chronic rejection morphological confirmation presented at biopsy already established severe graft injury (low eGFR, higher proteinuria), longer followup, higher expression of CDC20, CXCL6, DIABLO, GABRP, KIAA0101, ME2, MMP7, NFATC4, and TGFB3 mRNA, and lower expression of CCL19 and TRADD mRNA. In conclusion, both Banff 2007 chronic rejection categories did not differ in intrarenal expression of 376 selected genes associated with immune response.

## 1. Introduction

Both acute and chronic rejections have been shown to affect the long-term outcome of kidney transplantation. Chronic rejection is thought to be associated with both cellular and humoral alloimmune responses [1]. Chronic active antibody mediated rejection (CAMR) is characterized by C4d deposition in peritubular capillaries, the presence of circulating anti-donor antibodies, and morphologic evidence of chronic tissue injury such as glomerular double contours and peritubular capillary basement membrane multilayering and interstitial fibrosis/tubular atrophy (IF/TA) and fibrous arterial intimal thickening. The diagnosis of this entity is problematic since C4d deposits are not permanent and antibody mediated rejection was described to be associated also with different pathways where C4d is not involved [2]. Similarly, the chronic T-cell mediated rejection, albeit well described at Banff scheme, is of unclear pathogenesis. Moreover, the therapy of both processes remains to be insufficient. 

Beside conventional morphological evaluation, molecular histology offers better insight into rejection pathogenesis and prognosis. Moreover, molecular phenotype may better predict the graft outcome [3, 4].

In this study we aimed for evaluation of molecular signatures of acute and chronic rejections categories and for evaluation of association of gene transcripts with kidney graft loss due to chronic rejection. 

## 2. Materials and Methods

### 2.1. Patients

For the purpose of this study, 41 case biopsies revealing early acute AMR (*n* = 9), early acute T-cell mediated rejection (TCMR) (*n* = 10), chronic AMR (*n* = 13), and chronic TCMR (*n* = 9) performed in 2007–2009 were evaluated. Basic demographic parameters of patients are shown in Table 1. All patients were treated with maintenance immunosuppression based on either tacrolimus (TAC, 82%) or cyclosporine A (CsA, 10%), along with mycophenolate mofetil and corticosteroids, or using mTOR inhibitors (5%) or CNI with azathioprine (3%). Patients received induction therapy with rATG (Thymoglobulin, Genzyme) or daclizumab (Zenapax, Roche) in a case of PRA > 50% and 20%, respectively. All patients were followed up for at least 24 months after the biopsy. Graft failure was defined as a return to dialysis treatment. All patients gave their written informed consent to participate in the study, and the Ethics Committee of the Institute for Clinical and Experimental Medicine in Prague approved the study protocol. 

### 2.2. Renal Biopsy

All biopsies were performed using a 14-gauge Tru-Cut needle (Uni-Cut Nadeln, Angiomed, Germany) guided by ultrasound (Toshiba, Power Vision 6000, Japan). Small portions of renal tissue from the cortical or juxtamedullary zone were immediately stored in preserve solution (RNA later, Qiagen) for expression analysis, while the majority of renal tissue taken by core biopsy was used for routine histology performed by the standard method. Samples were routinely stained according to the protocol of our laboratory (H&E, PAS, Sirius red with elastin, AFOG, and PASM). Immunofluorescence detection of C4d was performed in all cases. Biopsy tissue was scored on the basis of the Banff '07 working classification [1]. 

### 2.3. RNA Isolation and Gene Expression Analysis

The renal tissue was homogenized. Total RNA was extracted by RNA Blue (Top-Bio) and reversely transcribed into cDNA, using the SuperScript II Reverse Transcriptase (Invitrogen). Complementary DNA samples from each biopsy were analyzed on TaqMan Low Density Array Cards containing primers and probe sets for targets by 7900HT Fast Real-Time PCR System (Applied Biosystems). The set of targets was chosen on the basis of potential relevance to the study of renal allograft rejection according to the existing literature data (see Supporting Table S1 available online at http://dx.doi.org/10.1155/2013/509259). Specific gene expression was calculated relative to that of the house-keeping gene glyceraldehyde-3-phosphate dehydrogenase (GAPDH) and the calibrator sample (FirstChoice Human Kidney Total RNA, Ambion) by comparative threshold cycle method (2^−ΔΔC_T_^). RQ Manager 1.2 software for automated data analysis (Applied Biosystems) was used and results were expressed as relative quantity (RQ). 

### 2.4. Statistical Analysis

After gene expression data were collected and the number of missing values was assessed. Two low quality samples with less than 50% of successfully measured genes were excluded from all other analyses. Similarly, genes successfully measured in less than 45% of biopsy samples were excluded from all other analyses. Gene expression was statistically analyzed for the remaining 305 genes. 

Basic statistical parameter data are given as absolute or relative frequency, average and SD, or median and range. Nonparametric test was used for analyzing data because of non normal distribution. Differences in mRNA expression or clinical parameters between groups were analyzed using the Kruskal-Wallis test and pairwise comparisons with Holm-Sidak correction and chi-square test for discrete variables. The differences in gene expression between groups expressed as RQ were considered to be biologically significant, if their means ratio reaches at least 1.5. PASW Statistics 18 software was used for statistical analyses. Unsupervised hierarchical clustering was performed using MeV software. Variables which can assess the graft outcome were defined by discriminant function analysis. The variables which were significantly different between failed and survived grafts with chronic AMR and chronic TCMR and had no missing values were included in that analysis after log transformation. A *P* value < 0.05 was considered to be statistically significant in all tests. 

## 3. Results

### 3.1. Patient Data

The clinical characteristics of patients are listed in Table 1. Patient age, gender, HLA mismatches, maximal or actual panel reactive antibodies (PRA), ischemia time, and number of failed grafts during 24 months followup did not differ significantly among patient groups. There were significantly more patients with retransplantation in the AMR group compared to others (*P* < 0.05). Significantly more patients received ATG induction therapy in AMR group as compared to other groups. Followup to biopsy was longer in acute AMR as compared to acute TCMR (*P* < 0.05); however, no differences were observed between chronic AMR and chronic TCMR. 

### 3.2. Gene Transcripts in Acute and Chronic Rejections

**Figure 1:**
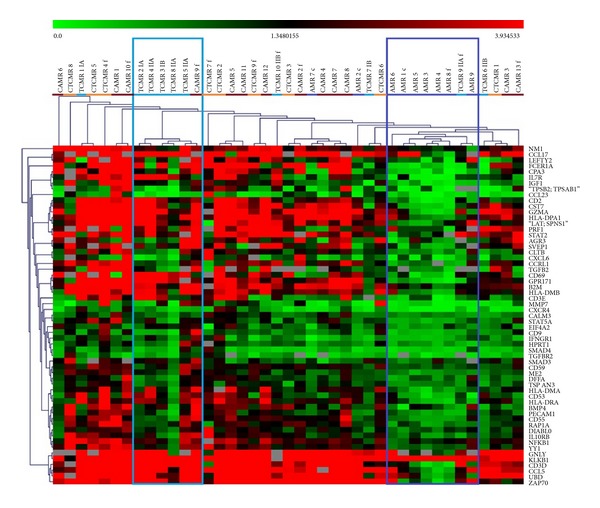
Unsupervised hierarchical clustering of samples with acute or chronic allograft rejection. Marked clusters contain 50% of TCMR samples or 78% of AMR samples, respectively. AMR samples out of the marked cluster had combined AMR and TCMR histological findings. Samples with CAMR and TCMR were mixed in different clusters. “f” in the sample name means failed; “c” in the sample name means combined AMR with TCMR.

**Figure 2:**
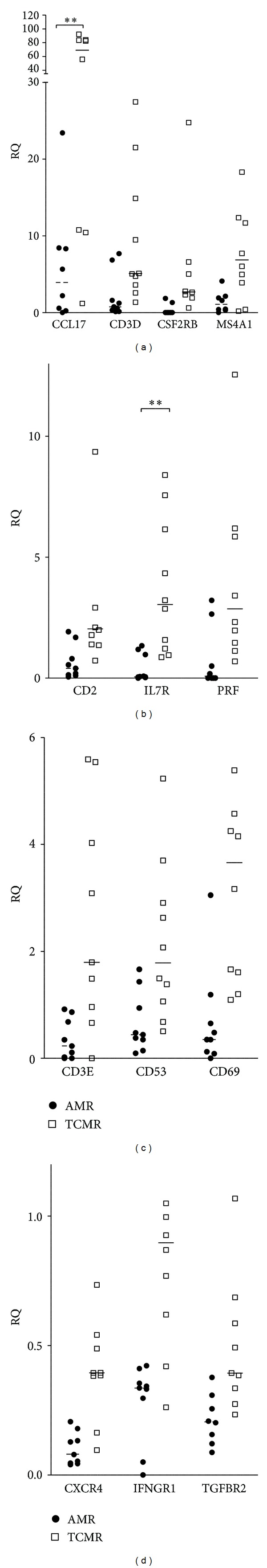
Genes differentially expressed between AMR and TCMR. Lines show medians of RQ. **P < 0.01; all unmarked plots: *P* < 0.05.

We compared the molecular profile among all rejection groups. In hierarchical clustering, samples with AMR were clustered in one cluster, except for two samples in which the AMR was combined with TCMR (Figure 1). The cluster included only one sample with different histological diagnoses (TCMR, type IIA). Fifty percent of samples with TCMR were grouped together in another cluster. The rest of samples with TCMR (majority with type IB or IIB) were mixed together with chronic rejection samples. Cluster analysis did not distinguish chronic AMR from chronic TCMR. Similarly, the pairwise comparison did not reveal any difference in gene expression between chronic AMR and chronic TCMR. On the contrary, in acute rejection the set of genes that were differently regulated in acute TCMR and acute AMR was identified (Figure 2).

### 3.3. Chronic Rejection Outcome Prediction

**Figure 3:**
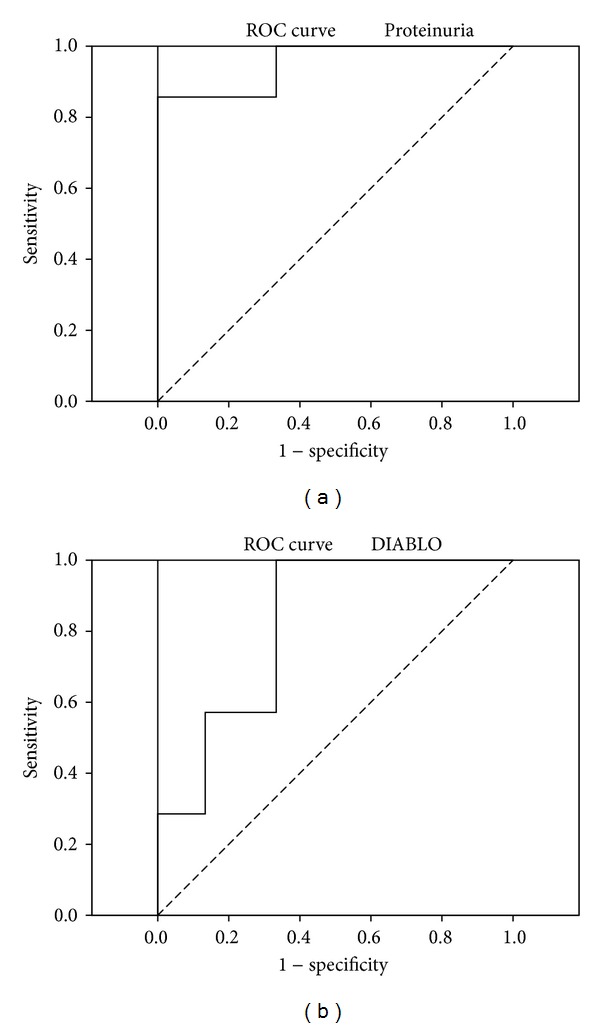
ROC curve analyzing graft failure after CAMR and CTCMR dependent on proteinuria at the time of biopsy (a) or intrarenal expression of DIABLO (b).

The effect of gene expression patterns on the chronic rejection outcome was analyzed. There were no differences in expressions of evaluated genes between chronic AMR and chronic TCMR. In 7 cases, graft function deteriorated during the 24-month followup after biopsy to CKD5T and dialysis therapy was initiated. These patients exhibited significantly worse renal function and proteinuria at the biopsy. Similarly, they had longer followup after transplantation, higher expression of CDC20, CXCL6, DIABLO, GABRP, KIAA0101, ME2, MMP7, NFATC4, and TGFB3 mRNA, and lower expression of CCL19 and TRADD mRNA (Table 2). All patients whose grafts failed as a consequence of chronic rejection underwent first renal transplantation. All different variables between failed and survived grafts after chronic rejection diagnosis were included in discriminant function analysis. This analysis revealed the proteinuria at the biopsy and DIABLO mRNA expression to discriminate failed and survived grafts. Next, classification functions containing these variables classified 100% of samples correctly. ROC analysis confirmed that proteinuria and DIABLO can predict the graft failure after chronic AMR or chronic TCMR (Figure 3, Table 3). 

## 4. Discussion

In this study, both acute and chronic rejections were shown to be associated with several patterns of immune system related gene transcripts. Recently, despite different pathologies, both T-cell mediated and antibody mediated rejections were shown to be associated with similar molecular features [2, 5]. In both rejection types, interferon gamma (IFNG) related gene transcripts were shown to play a role. IFNG-inducible or cytotoxic T-cell associated transcripts distinguished rejection from nonrejection and were elevated in both acute T-cell mediated and antibody mediated rejections [6]. In our study, the upregulation of a large set of immune system related genes in acute T-cell mediated rejection was observed. Of note, IFNG-inducible and cytotoxic T-cell associated transcripts were upregulated in the acute TCMR that is in line with the observation of others [5, 6]. However, gene transcripts in both chronic rejection types were similar in our study. Neither paired test nor hierarchical clustering distinguished these types of chronic rejection on the molecular basis. There are few data in the current literature dealing with gene expression in chronic rejection of renal allograft. Changes in histological classification that passed off in the last several years could have contributed to the lack of such studies. While in Banff '97 classification [7] the chronic changes were represented by non specific chronic allograft nephropathy category, Banff '05 Meeting substituted it by interstitial fibrosis and tubular atrophy (IF/TA) and the terms chronic active antibody mediated rejection and chronic active T-cell-mediated rejection were included [8]. Another complication seems to be the relative rarity of biopsies diagnosed as chronic active T-cell-mediated rejection. Studies dealing with molecular phenotyping of chronic antibody mediated rejection focused on comparison with samples from patients with stable renal function and normal histopathology [9]. However, our study is one of the first studies focusing on comparison of gene expression in precisely defined histological findings, described by Banff 2007 classification as antibody-mediated and T-cell-mediated chronic rejections. It was not possible to find out differences between those two diagnoses without prior whole-genome microarray screening only on the basis of the literature-based selection of 305 analyzed genes. Several genes indicating inflammation were significantly upregulated in grafts that failed after chronic rejection in our study. Other upregulated genes in failed grafts belonged to cytotoxic T cells associated transcripts, ENDATs, chemotactic transcripts, or apoptosis markers. Some of them were referred to predict graft failure also in the study of Einecke et al. [10]. In the discriminant function analysis, however, the outcome of chronic rejection in our cohort of patients depended only on the state of disease and graft injury (proteinuria) in combination with intrarenal expression of DIABLO, the caspase activator playing the key role in apoptosis. It was clearly shown that proapoptotic mechanisms have been implicated in ischemia induced acute kidney injury [11]. Similarly, chronic rejection of kidney allograft is associated with small vessels narrowing causing local ischemia. 

In conclusion, in this study, beside transcripts differences observed between acute T-cell mediated and antibody mediated rejections, both chronic rejections did not differ in 305 analyzed genes. 

## Supplementary Material

The list of 345 genes evaluated by real-time quantitative PCR. The set of targets was chosen on the basis of potential relevance to the study of renal allograft rejection according the existing literature data.

## Figures and Tables

**Table 1:** Basic patient characteristics.

	AMR^a^	TCMR^b^	CAMR	CTCMR
*N *	9	10	13	9
Age	39.56 ± 9.91	49.63 ± 13.96	50.13 ± 11.63	52.60 ± 14.80
Female gender	1 [11.1%]	2 [20.0%]	5 [38.5%]	3 [33.3%]
HLA mismatches: total	4.0 ± 1.3	3.6 ± 1.1	3.4 ± 1.2	3.1 ± 1.1
HLA-A	1.4 ± 0.5	1.2 ± 0.7	1.2 ± 0.7	1.0 ± 0.5
HLA-B	1.5 ± 0.8	1.6 ± 0.5	1.3 ± 0.6	1.2 ± 0.4
HLA-DR	1.1 ± 0.6	0.8 ± 0.4	1.0 ± 0.6	0.9 ± 0.6
PRA at Tx [%]	17.4 ± 22.0	17.3 ± 36.0	17.5 ± 21.9	13.7 ± 12.1
IS: triple therapy based on FK	9 [100%]	10 [100%]	7^c^ [53.8%]	8 [88.9%]
triple therapy based on CsA	0 [0.0%]	0 [0.0%]	4 [30.8%]	0 [0.0%]
mTORi	0 [0.0%]	0 [0.0%]	1 [7.7%]	1 [11.1%]
other	0 [0.0%]	0 [0.0%]	1 [7.7%]	0 [0.0%]
First/second/third/fifth transplants	1/6/1/1^d^	8/2/0/0	8/4/1/0	7/1/1/0
Induction therapy	9 [100%]	3 [30.0%]	5 [38.5%]	3 [33.3%]
Time to biopsy (months)			91.81 ± 66.99^e^	33.84 ± 46.65^e^
(days)	12 ± 4^f^	8 ± 3^f^		
sCr at Bx (*µ*mol/L)	358.07 ± 148.55	398.66 ± 210.44	213.47 ± 105.15	272.99 ± 98.74
eGFR at Bx (mL/s/1.73 m^2^)	0.34 ± 0.16	0.36 ± 0.26	0.49 ± 0.22	0.34 ± 0.11
Proteinuria at Bx (g/day)	1.77 ± 1.43	2.26 ±1.94	2.8 ± 3.9	1.3 ± 1.1
C4d +	9 [100%]	0 [0.0%]	13 [100%]	0 [0.0%]
Graft loss during the followup (*n*)	1 [11.1%]	2 [20.0%]	4 [30.8%]	3 [33.3%]

Continuous variables are means ± SD.

^a^Including combined AMR and TCMR (*n* = 3).

^b^Type IA (*n* = 2), IIA (*n* = 4), IB (*n* = 2), and IIB (*n* = 2).

^c^Significantly fewer FK treatment than in other groups (*P* < 0.05).

^d^Significantly more retransplantation in AMR (*P* < 0.05).

^e^No significant difference between CAMR and CTCMR.

^f^Significantly longer time to rejection in AMR compared to TCMR (*P* < 0.05).

**Table 2:** Significant differences between failed and survived grafts after chronic rejection (both CAMR and CTCMR). Only variables that reach statistical significance are listed.

	Failed grafts (*n* = 7)	Survived grafts (*n* = 15)	*P *
sCr at Bx [*µ*mol/L]	298.1 [197.3–456.8]	193.5 [91.2–485.6]	0.005
eGFR at Bx [mL/s/1.73 m^2^]	0.28 [0.18–0.37]	0.47 [0.13–0.98]	0.008
Proteinuria at Bx [g/day]	3.47 [0.61–12.58]	0.47 [0–2.35]	0.001

CCL19	1.5197 [0.1042–11.1551]	8.6061 [0.4848–82.2367]^b^	0.044
CDC20	2.4168 [1.8262–3.1978]	0.9337 [0.4203–3.0410]	0.007
CXCL6	2.6814 [0.3292–14.7608]^a^	0.4933 [0.0000–1.9457]^b^	0.039
DIABLO	1.9110 [1.6832–6.2070]	1.3873 [0.7627–2.0413]	0.018
GABRP	3.5970 [0.5333–13.8507]^a^	0.2527 [0.0000–7.2719]^b^	0.013
KIAA0101	5.9674 [2.8230–17.7875]	3.7627 [0.2982–6.2863]	0.032
ME2	1.7962 [1.0032–2.5815]	1.1111 [0.6937–2.5290]	0.038
MMP7	1.4519 [0.8888–4.4876]	0.4838 [0.0026–4.3613]	0.004
NFATC4	2.3609 [0.8072–7.0016]^c^	0.7314 [0.0000–2.3032]^b^	0.012
TGFB3	1.1726 [0.1405–2.0126]^c^	0.1292 [0.0000–15.6462]^d^	0.034
TRADD	2.3305 [0.9925–16.0450]^c^	9.6845 [3.1361–45.8793]^e^	0.037

Variables are presented as median (min–max).

^a^ *n* = 6.

^b^ *n* = 14.

^c^ *n* = 5.

^d^ *n* = 12.

^e^ *n* = 10.

**Table 3:** Cutoff values form ROC curve analysis that discriminate failed and survived grafts with the best combination of sensitivity and specificity.

	Optimal cutoff	Sensitivity	Specificity	AUC	95% CI
Proteinuria	2.35	85.7	100	0.952	0.768–0.993
DIABLO	1.43	100	66.7	0.819	0.598–0.947
