# Supplementary material for: Molecular Profiling of Acute and Chronic Rejections of Renal Allografts
Source: Clin Dev Immunol. 2013 Nov 4;2013:509259. doi: 10.1155/2013/509259 (PMC3834978; doi:10.1155/2013/509259)
Supplement: Supplementary file 1 — The list of 345 genes evaluated by real-time quantitative PCR. The set of targets was chosen on the basis of potential relevance to the study of renal allograft rejection according the existing literature data. [file 509259.f1.pdf]

SUPPORTING TABLE S1: Genes evaluated by real-time quantitative PCR

ADAMTSL3	CALM2	CCRL2	CSF2	GDF6	IL15	LAT;SPNS1	PDGFB	TGFB1	XCR1
AGR2	CALM3	CD14	CSF2RB	GDF9	IL16	LBP	PECAM1	TGFB2	YY1
AGR3	CASP1	CD2	CST7	GNLY	IL17A	LCK	PFN2	TGFB3	ZAP70
AKT1	CASP10	CD27	CTLA4	GPR171	IL18	LEFTY1	PPIF	TGFBR1	
ANXA5	CASP2	CD28	CTNNB1	GZMA	IL18R1	LEFTY2	PRF1	TGFBR2	
ANXA7	CASP3	CD3D	CX3CL1	GZMB	IL1A	LTA	PRL	TGFBR3	
APAF1	CASP6	CD3E	CX3CR1	GZMH	IL1B	LTB	PRLR	TLR1	
APOA2	CASP7	CD3G	CXCL1	GZMK	IL1R1	LTBP1	PTGS2	TLR2	
APOH	CASP8	CD4	CXCL10	HIF1A	IL1R2	LTBP2	PTPRC	TLR3	
ATF1	CASP9	CD40	CXCL11	HLA-C	IL1RAP	LTBP4	PTTG1	TLR4	
ATF2	CCL1	CD40LG	CXCL12	HLA-DMA	IL2	LY96	RAP1A	TLR5	
ATF3	CCL11	CD53	CXCL13	HLA-DMB	IL23A	LYZ	RB1	TLR6	
ATP1A1	CCL13	CD55	CXCL2	HLA-DPA1	IL24	MANEA	RBL2	TLR7	
ATP5B	CCL16	CD59	CXCL3	HLA-DQA1	IL2RA	MAP3K5	REL	TLR9	
ATP5G3	CCL17	CD69	CXCL5	HLA-DQA2	IL2RB	MAPK8	RELA	TNF	
B2M	CCL18	CD70	CXCL6	HLA-DRA	IL3	ME2	RELB	TNFRSF10A	
BAD	CCL19	CD74	CXCL9	HLA-E	IL4	MMP2	RGN	TNFRSF10B	
BATF	CCL2	CD79A	CXCR3	HMOX1	IL4R	MMP7	RGS13	TNFRSF11B	
BAX	CCL21	CD79B	CXCR4	HRSP12	IL5	MMP9	RRM2	TNFRSF18	
BCL2	CCL22	CD80	CXCR6	CHODL	IL6	MPL	SERPINA3	TNFRSF1A	
BCL2L11	CCL23	CD81	CYCS	ICAM1	IL6R	MS4A1	SMAD1	TNFRSF1B	
BDNF	CCL24	CD86	DFFA	ICOS	IL7	MYD88	SMAD2	TNFRSF21	
BFAR	CCL3	CD8A	DIABLO	ICOSLG	IL7R	NCK1	SMAD3	TNFRSF25	
BID	CCL4	CD9	EEF1A1	IFNA1	IL8	NCK2	SMAD4	TNFRSF9	
BMP1	CCL5	CDC20	EFHD1	IFNB1	IL8RA	NEGR1	SMAD5	TNFSF10	
BMP2	CCL7	CDH17	EIF4A2	IFNG	IL8RB	NEK2	SMAD9	TNFSF14	
BMP3	CCL8	CDK2AP1	FABP1	IFNGR1	IL9	NFAT5	SMURF1	TNFSF4	
BMP4	CCNA2	CEBPB	FADD	IFNGR2	IL9R	NFATC1	SNAP25	TOLLIP	
BMP5	CCNB1	CFLAR	FAS	IFNK	INHA	NFATC2	SOCS1	TP53	
BMP6	CCNB2	CKS1B	FASLG	IGF1	INHBA	NFATC3	SOCS2	TP53BP2	
BMP7	CCR1	CKS2	FCER1A	IGF1R	ITGA2	NFATC4	SOCS5	TPSB2;TPSAB1	
BMP8B	CCR10	CLEC7A	FIGF	IGF2	ITGB2	NFKB1	SPP1	TRADD	
BRAF	CCR2	CLTB	FOS	IGLL1	ITK	NLGN4X	STAT1	TRAF2	
C1S	CCR3	COL1A1	FOXP3	IL10	JAK1	NMI	STAT2	TRAF3	
C3	CCR4	COL1A2	GABRP	IL10RA	JAK2	NOS3	STAT3	TSPAN1	
C3AR1	CCR5	COL3A1	GATM	IL10RB	JUN	ORM2	STAT4	TSPAN3	
C4A;C4B	CCR6	CPA3	GBP1	IL12A	JUNB	OXCT1	STAT5A	TYK2	
C7	CCR7	CREB1	GCA	IL12B	KIAA0101	PAPLN	STAT5B	UBD	
